# Is PrEP necessary during natural conception in HIV-1-serodiscordant couples on ART with suppressed viral load? A retrospective cohort analysis

**DOI:** 10.1186/s12879-020-4912-4

**Published:** 2020-03-06

**Authors:** Lijun Sun, An Liu, Jianwei Li, Ying Shao, Qiuyun Li, Jiangzhu Ye, Hongwei Zhang, Zaicun Li, Hui Wang

**Affiliations:** 1 Center for Infectious Diseases, Beijing Youan Hospital, Capital Medical University, Beijing, 100069 China; 2 Department of Infectious Diseases, Shenzhen Third People’s Hospital, Shenzhen, 518112 China

**Keywords:** HIV, PrEP, Viral suppression, ART

## Abstract

**Background:**

Antiretroviral therapy (ART) demonstrates high efficacy in reducing the risk of HIV transmission to sexual partners. However, it is not clear if the use of pre-exposure prophylaxis (PrEP) in HIV-1-serodiscordant couples is necessary during natural conception when the HIV-positive partner exhibits a suppressed viral load. The purpose of this study was to assess the role of PrEP during natural conception in this population.

**Methods:**

A retrospective, multicenter study was conducted in a cohort of HIV-1-serodiscordant couples (positive man, negative woman) with childbearing desires. HIV-positive male partners were treated with ART and achieved viral suppression for more than half a year. The HIV-negative female partners were either treated with PrEP or not treated with PrEP, and outcomes were compared between the two treatment groups.

**Results:**

Of 246 HIV-1-serodiscordant couples in whom the HIV-positive partner achieved viral suppression, 104 seronegative women were treated with PrEP during natural conception and 142 seronegative women were not treated with PrEP. There were 410 condom-less sexual acts in couples treated with PrEP and 615 condom-less sexual acts in couples not treated with PrEP. We observed no instances of HIV transmission in HIV-1-serodiscordant couples with or without the use of PrEP during the process of natural conception.

**Conclusions:**

Our results show that PrEP had minimal influence in reducing the risk of HIV transmission during natural conception in HIV-1-serodiscordant couples with a stably suppressed viral load. Thus, it may be an acceptable option for HIV-negative partners to not use PrEP during the process of natural conception if the HIV-positive partner has achieved viral suppression for more than half a year.

## Background

Antiretroviral therapy (ART) has not only significantly reduced the risk of sexual transmission of the human immunodeficiency virus (HIV), but has also dramatically improved the life expectancy in people living with HIV [1, 2]. A study in HIV-1-serodiscordant couples showed that compared to delayed ART, early initiation of ART among HIV-positive partners was associated with a 93% reduction in the risk of infection in HIV-negative partners [3]. Moreover, another study found that HIV-infected persons who were treated with ART and achieved viral suppression were 94% less likely to transmit HIV compared to persons with undiagnosed HIV [4]. Further studies showed that the risk of HIV transmission through sex when HIV viral load is suppressed is effectively zero without pre-exposure prophylaxis (PrEP) in serodiscordant couples [5, 6].

In developing countries such as China, reproductive options for HIV-serodiscordant couples are limited in that intrauterine insemination and in vitro fertilization can be expensive or are not accessible to HIV-infected people. With the effectiveness of ART in reducing the risk of sexual transmission, natural conception has become a relatively safe and exciting reproductive option for HIV-1-serodiscordant couples. In addition to timed intercourse and sustained viral suppression in HIV-positive partners, PrEP is used in HIV-negative partners to reduce the risk of HIV infection during the process of natural conception [7, 8].

Recent studies have indicated that linked HIV infections were not observed in serodiscordant couples having condom-less sex when the HIV-positive partner exhibited a stably suppressed plasma viral load [9, 10]. As such, natural conception appears to be a realistic and safe option for conceiving a child in this population. In HIV-serodiscordant couples in which the woman is HIV-positive, semen is injected into the vagina with a syringe, and thus, the HIV-negative male partner has no risk of contracting HIV. However, in HIV-serodiscordant couples in which the man is HIV-positive, the natural conception process comes with risks. In the present study, we assessed outcomes in HIV-1-serodiscordant couples (HIV-positive man) who did or did not use PrEP during natural conception.

## Methods

### Study population

This retrospective study examined the medical records of HIV-1-serodiscordant couples who were treated from January 2008 to January 2018 at sexually transmitted disease/acquired immunodeficiency syndrome clinics of Beijing Youan Hospital, Capital Medical University and the Third People’s Hospital of Shenzhen. All couples expressed a desire to conceive and were given general HIV information as well as information about the risk of transmitting HIV during natural conception. They were also told that it was unclear whether PrEP provided any additional benefit in reducing HIV transmission between partners in couples with a virally suppressed HIV-positive partner. The choice to use or not use PrEP was voluntary. The couples gave informed consent to have data extracted from their medical records. Ethical approval was obtained from the Ethics Committee of Beijing Youan Hospital, Capital Medical University.

### Preparation for fertilization

Preparation for fertilization was done as previously described [7]. Briefly, HIV-negative female partners underwent screening for infection and a gynecologic examination. To monitor ovulation, basal body temperatures were measured, and urine luteinizing hormone levels were evaluated with a urine luteinizing hormone ovulation test kit (Gemc Technology Co., Ltd., Zhengzhou, China). For some women who were not suitable for basal body temperature and whose test strip assay for ovulation was not ideal, an ovary ultrasound was used to monitor ovulation.

The HIV-positive male partner in the HIV-1-serodiscordant couples was treated with ART. Natural conception was advised in couples whose positive-partner exhibited sustained undetectable plasma viral load (< 50 copies/ml) for at least 6 months. Unprotected sexual relations were limited to the periovulatory period (timed intercourse).

### Intervention of HIV horizontal transmission

The HIV-1-discordant couples were divided into those who seronegative female partners were treated with PrEP and those whose seronegative female partners were not treated with PrEP. In the PrEP-treated group, zidovudine (AZT) + lamivudine (3TC), tenofovir/emtricitabine (TDF/FTC), TDF/FTC + raltigravir (RAL) or lopinavir/ritonavir (LPV/r)-based regimens (TDF/FTC, TDF + 3TC, and AZT + 3TC) was administrated to the seronegative female partner starting the morning of luteinizing hormone-peak until 28 days after the last instance of condom-less intercourse. The antiretrovirals used for PrEP were chosen based on availability. The seronegative female partners were tested for HIV infection at 6, 12, and 24 weeks after the last occurrence of condom-less intercourse. After successful conception, the couples resumed condom use during sexual activities, and PrEP antiretroviral use ceased.

### Statistical analysis

A descriptive analysis of the sociodemographic and clinical characteristics of the HIV-1-serodiscordant couples was performed. We analyzed the epidemiological characteristics, clinical profile of the HIV-positive partners, total number of unprotected acts of vaginal intercourse, and risk of horizontal transmission. We compared characteristics and outcomes between PrEP use and non-PrEP use groups using the chi-square or Mann-Whitney U test and stratified by sex. The number of vertical transmissions was recorded. A *p*-value < 0.05 indicated statistical significance. All data were analyzed with SPSS version 17.0 (SPSS, Chicago, IL, USA).

## Results

A total of 246 HIV-1-serodiscordant couples (positive man, negative woman) were included in this study. Of the 246 couples, 104 seronegative women were treated with PrEP. The remaining 142 seronegative women were not treated with PrEP. The characteristics of the couples are shown in Table 1. The PrEP regimens most commonly used in this study were TDF/FTC (25 cases), TDF/FTC + RAL (52 cases), and LPV/r-based regimens [TDF/FTC (9 cases), TDF + 3TC (9 cases), and AZT + 3TC (6 cases)]. Only three cases received the regimen of AZT + 3TC for PrEP.

**Table 1:** Characteristics of HIV-1-serodiscordant couples in this study

	Cohort, No. (%)		*P*-value
	PrEP group (*n* = 104)	Non-PrEP group (*n* = 142)	
Seronegative woman			
Age, median (IQR), y	28 (23, 30)	27 (24, 31)	0.999
Native place			0.997
East China	31 (29.8)	43 (30.3)	
Central China	11 (10.6)	15 (10.6)	
West China	62 (59.6)	84 (59.1)	
Education level			0.518
Bachelor’s degree or above	46 (44.2)	54 (38.0)	
High school and vocational school	17 (16.4)	30 (21.1)	
Middle school or below	41 (39.4)	58 (40.9)	
Seropositive man			
Age, median (IQR), y	30 (26, 35)	30 (27, 35)	0.552
Transmission route			0.707
Male-to-male sexual contact	85 (81.7)	108 (76.1)	
Heterosexual contact	9 (8.7)	15 (10.6)	
Bisexual	5 (4.8)	8 (5.6)	
Blood transmission	2 (1.9)	7 (4.9)	
Unknown	3 (2.9)	4 (2.8)	
Regimens			0.063
LPV/r based	3 (2.9)	11 (7.8)	
RAL based	0 (0.0)	1 (0.7)	
EFV based	94 (92.2)	126 (88.7)	
Others	5 (4.9)	4 (2.8)	
Treatment duration (IQR), y	3.5 (2.7, 4.5)	4.2 (3.3, 5.7)	0.003
Baseline CD4, cell/μl	543 (442, 637)	560 (426, 690)	0.537

*HIV* human immunodeficiency virus, *PrEP* pre-exposure prophylaxis, *IQR* interquartile range, *LPV/r* lopinavir/ritonavir, *RAL* raltegravir, *EFV* efavirenz

A total of 410 condom-less sexual acts were reported for the couples that received PrEP whereas 615 condom-less sexual acts were reported for the couples that did not receive PrEP. Seroconversion did not occur in either group of HIV-1-serodiscordant couples during natural conception. The number of successful pregnancies was 104 in the PrEP group and 142 in the non-PrEP group (Table 2).

**Table 2:** Comparison of reported sexual acts between HIV-1-serodiscordant couples with or without PrEP

	PrEP group (*n* = 104)	Non-PrEP group (*n* = 142)	*P*-value
Condom-less sex act(s)			0.217
1	6	19	
2	23	13	
3	27	27	
4	9	16	
5	18	32	
6	7	13	
7	9	12	
8	4	3	
9	1	2	
10	0	3	
12	0	2	
Mean of ovulation cycle	2.65	2.67	0.847
Seroconversion	0	0	
Pregnancies	104	142	

## Discussion

PrEP has been shown to be an effective option for preventing the transmission of HIV. A previously published study reported a 99% reduction in the risk of HIV infection when PrEP was taken 7 days a week [11]. A sub-study within the Partners PrEP study analyzed adherence to antiretroviral prophylaxis among African HIV-serodiscordant couples and found that the efficacy of PrEP was 100% among highly adherent PrEP users [12].

Natural conception is becoming an acceptable option for some HIV-serodiscordant couples in resource-limited settings, since reproductive technologies such as in vitro fertilization and intracytoplasmic sperm injection with prepared sperm are either too expensive or not available to HIV-infected people [7]. With condom-less intercourse limited to periods of fertility, “PrEP for conception” is safe, effective, and a Centers for Disease Control and Prevention-endorsed conception strategy for serodiscordant couples [13, 14]. However, some couples may not choose PrEP due to economic reasons or may worry about the drugs’ side effects.

Our study analyzed the use of PrEP during natural conception in HIV-1-serodiscordant couples in which the viral load in the HIV-infected partner was suppressed. Our results showed that there were no cases of HIV seroconversion in uninfected sexual partners in both the PrEP use and non-PrEP use groups. This suggests that natural conception is relatively safe without PrEP use when timed intercourse is employed in HIV-1-serodiscordant couples with a virally suppressed HIV-positive partner. Natural conception without PrEP has also been analyzed in other studies [8, 15].

In 1994, a study showed that HIV-infected men treated with zidovudine monotherapy were half as likely to transmit infection to their female partners compared to untreated men [16]. In 2009, a meta-analysis identified 11 cohorts reporting on 5021 heterosexual couples and found that there were no instances of HIV transmission in patients that were treated with ART and had a viral load below 400 copies/ml [1]. Several studies have recently indicated that linked HIV transmission was not observed in serodiscordant couples who had condom-less sex if the HIV-positive partner had a stably suppressed plasma viral load [5, 9, 10]. As a result of these groundbreaking findings, the message that HIV-positive individuals with an undetectable viral load could not transmit the virus to their partners was widely circulated. The slogan “Undetectable = Untransmittable” (U = U) was popularized by the Prevention Access Campaign, which was supported by 819 organizations from 97 countries [17].

The effectiveness of ART in preventing HIV transmission is dependent upon an HIV-positive individual maintaining full virological suppression in plasma. Once an individual on ART is virally suppressed and maintains good drug adherence, the risk of viral load rebound is very low. The PARTNER 2 study [6] which included 782 serodifferent, same sex couples that were followed for almost 1600 eligible couple-years found zero instances of within-couple HIV transmission.

Our findings provide new evidence for the “U = U” message and support the concept that HIV-positive people who are adhering to treatment and exhibit a suppressed viral load do not transmit HIV to HIV-negative sexual partners. The message has allowed HIV-serodiscordant couples to engage in condom-less sexual acts without fear. Furthermore, it can reduce the burden of drug use during pregnancy without PrEP and reduce the potential risk of the drugs. However, in some cases, couples are reluctant to disclose their HIV or serodiscordant relationship status to families or communities and feel constrained to rely on each other for social support in coping with HIV. PrEP offers potential relief from stress and may increases trust within the relationship. Recognizing the issues these couples face enables physicians to offer honest and nonjudgmental preconception counseling [14].

There were several limitations to our study. First, this study was conducted retrospectively by case selection and was not randomized, so there was the potential of selection bias. Second, the number of HIV-serodiscordant couples was small. A larger prospective study should be conducted to evaluate the efficacy of PrEP use during natural conception among HIV-serodiscordant couples.

## Conclusions

In summary, our study examined whether PrEP use during natural conception was beneficial in reducing the risk of HIV transmission among HIV-1-serodiscordant couples that have an HIV-positive partner who has achieved long-term viral suppression. Our study found no cases of HIV seroconversion in the PrEP group and the non-PrEP group. Our results indicate that PrEP use during natural conception may not be necessary in HIV-1-serodiscordant couples if the HIV-positive partner has stably suppressed plasma viral loads. Furthermore, our findings provide new evidence for the message of “U = U.” The decision to undergo PrEP during the process of natural conception in HIV-serodiscordant couples should be based on sustained viral suppression in the HIV-positive partner and acceptance of “U = U” in the HIV-negative partner.

## Data Availability

The datasets used and/or analyzed during the current study are available from the corresponding author on reasonable request.

## Abbreviations

ART: antiretroviral therapy
HIV: human immunodeficiency virus
PrEP: pre-exposure prophylaxis
